# Systematic Design of 18S rRNA Gene Primers for Determining Eukaryotic Diversity in Microbial Consortia

**DOI:** 10.1371/journal.pone.0095567

**Published:** 2014-04-22

**Authors:** Luisa W. Hugerth, Emilie E. L. Muller, Yue O. O. Hu, Laura A. M. Lebrun, Hugo Roume, Daniel Lundin, Paul Wilmes, Anders F. Andersson

**Affiliations:** 1 KTH Royal Institute of Technology, Science for Life Laboratory, School of Biotechnology, Division of Gene Technology, Stockholm, Sweden; 2 Luxembourg Centre for Systems Biomedicine, University of Luxembourg, Esch-sur-Alzette, Luxembourg; King Abdullah University of Science and Technology, Saudi Arabia

## Abstract

High-throughput sequencing of ribosomal RNA gene (rDNA) amplicons has opened up the door to large-scale comparative studies of microbial community structures. The short reads currently produced by massively parallel sequencing technologies make the choice of sequencing region crucial for accurate phylogenetic assignments. While for 16S rDNA, relevant regions have been well described, no truly systematic design of 18S rDNA primers aimed at resolving eukaryotic diversity has yet been reported. Here we used 31,862 18S rDNA sequences to design a set of broad-taxonomic range degenerate PCR primers. We simulated the phylogenetic information that each candidate primer pair would retrieve using paired- or single-end reads of various lengths, representing different sequencing technologies. Primer pairs targeting the V4 region performed best, allowing discrimination with paired-end reads as short as 150 bp (with 75% accuracy at genus level). The conditions for PCR amplification were optimised for one of these primer pairs and this was used to amplify 18S rDNA sequences from isolates as well as from a range of environmental samples which were then Illumina sequenced and analysed, revealing good concordance between expected and observed results. In summary, the reported primer sets will allow minimally biased assessment of eukaryotic diversity in different microbial ecosystems.

## Introduction

The accurate identification of diversity is a key challenge in microbial ecology research. Molecular biology techniques, most notably polymerase chain reaction (PCR)-based amplification and sequencing of the resulting small-subunit ribosomal DNA amplicons, have revolutionized our view of microbial diversity by unveiling a tremendous diversity of bacteria, archaea and eukarya in different environments [1–4). Recent developments in high-throughput sequencing make deep sequencing of hundreds of samples achievable at affordable costs. This is in turn transforming microbial ecology into a quantitative research field in which, for example, models of spatio-temporal patterns of microbial diversity can be built and tested [5]–[11], and links between microbial community composition and host genotype [12] as well as phenotype [13]–[16] can be established. So far, massively parallel sequencing technology has mostly been applied for addressing bacterial and archaeal diversity. However, recently, 454 pyrosequencing has been conducted on 18S rDNA amplicons and revealed an unprecedented diversity of eukaryotes in a range of environments [17]–[26].

Current high-throughput sequencing platforms can generate billions of short reads, often below 200 bp. Accurate identification of microbial taxa with this limited amount of information demands a diligent choice of PCR primers, so as to simultaneously prioritize the sequencing of highly informative regions and avoid biases caused by unevenly amplifying different taxa. The size of the amplicon is also a limiting factor for most high-throughput technologies. Considerable efforts have been put into designing optimal primers for bacterial and archaeal identification [6]; [27]–[34]. However, despite the efforts of and Amaral-Zettler *et al*, Nolte *et al* and Stoeck *et al* [17], [36], [18], no equally broad and systematic design of eukaryotic primers has yet been conducted by leveraging the richness of sequences in current databases. In fact, several recent studies perform high-throughput sequencing while using primers never fully assessed for this, such as the ones in the pioneering work of Pace and Sogin [1], [35] or only assessed for DGGE [37]–[39]. Both these cases highlight the need for broad taxonomy-range primer pairs for the generation of information-rich amplicons.

Here, in an effort to develop 18S rDNA primer sets compatible with current high-throughput sequencing technologies and which should generate minimally biased, phylogenetically discriminatory sequences, we have used all non-redundant full-length 18S rDNA sequences from the SILVA database [40] to identify optimal primer pairs for partial 18S sequencing. We did this by first identifying a set of degenerate primers with high coverage among reference sequences and evaluating the taxonomic distribution of their matches. We then combined these into a set of candidate primer pairs for which we assessed *in silico* the phylogenetic information that would be generated with each primer pair when using different massively parallel sequencing technologies. One of the primer pairs that performed best *in silico* was optimised for PCR and subsequently used to amplify the V4-V6 region of the 18S rDNA sequences from a range of environments that were subsequently sequenced by Illumina technology.

The developed primer pairs are optimally suited for identifying eukaryotic taxa through 18S rDNA sequencing using read lengths typically obtained using massive parallel sequencing, which can be as short as pairs of 150 bp paired-end reads or 400 bp single-end reads, while still resulting in phylogenetically discriminatory data.

## Materials and Methods

### Degenerate Primer Design

The “SSU Ref NR” aligned sequences were downloaded from release 111 of the SILVA database (www.arb-silva.de; [40]), and eukaryotic sequences were extracted from these based on their annotation. After first trimming the alignment to only include positions represented by 90% of the sequences (while maintaining information on where nucleotides had been removed), the program DegePrime (https://github.com/EnvGen/DEGEPRIME; Hugerth *et al.* submitted) was used to generate 18 bases long degenerate primers at every alignment position, with maximum degeneracy 12. From these, the number of sequences from each taxon matched by the primers was counted. To generate the modified primers 574* and 616*, sequences corresponding to the taxa that were missed by primers 574 and 616, respectively, were extracted from the SILVA database and DegePrime was run on them. By comparing the resulting oligonucleotides, we identified the degeneracies that should be added to each primer and, in the case of position 574, where the 18^th^ nucleotide would be N, we opted to remove this base.

### *In Silico* Simulations

All reads were simulated from the “SSU Ref NR” database. Error free reads were simulated starting at each primer sequence(s), and extending for 150, 250 or 400 bases, with read length including primer length. When counting unique sequences, in case the paired reads for a primer pair did not span the entire amplicon, they were concatenated and, in case the distance between a primer pair was shorter than their combined read length, the overlapping ends were merged.

For the BLAST-based analyses, reads from 1000 unique sequences were selected at random, including only sequences of defined phylogeny matched by all candidate primers. Stand-alone BLASTN was used for matching reads to sequences in the original database. Hits were kept if they matched at least 95% of the read length with 95% or 99% identity, as stated in the results section, and with an e-value below 10E-5. In the case of paired-ended reads, the hit with the lowest combined e-value was kept. E-values were combined through the mean of their log e-values, which corresponds roughly to averaging their bit-scores. Taxonomic accuracy was assessed only in the cases where the selected hit was annotated at least to family level. Distances between full-length query and hit sequences were calculated on Mothur [41], disregarding terminal gaps from the original aligned SILVA file and treating each string of gaps as a single gap. All statistical analyses and graphs were produced in R, with the additional library Vioplots.

### DNA Extraction, Amplification and Sequencing

No animals were killed for the purpose of this study. Moose were shot by licensed hunters during Swedish hunting season in 2012. Rumen content was sampled post-mortem, for which no ethical permission is required under Swedish law. The human faeces sample was first used in Forberg’s *et al* study [42] and all permissions therein apply.

DNA fractions were extracted from wastewater and moose rumen (*Alces alces*) according to the protocol in Roume *et al* [43] and from soil and marine sediment using the PowerSoil kit (MO BIO Laboratories Inc, Carlsbad CA, USA). One marine sample was extracted using the PowerWater kit (MO BIO Laboratories Inc, Carlsbad CA, USA), the other as described by Riemann *et al* [44], and further purified by ethanol precipitation. The faecal sample was extracted with DNeasy Blood & Tissue kit (Qiagen, Venlo, Netherlands). Optimization of PCR conditions for selective amplification of 18S rDNA was initially carried out with DNA extracted from a pure culture of *Saccharomyces cerevisiae* for eukaryotes as positive control, *Escherichia coli* for bacteria and *Halobacterium* sp. NRC-1 for archaea as negative controls. The best temperature for primer annealing was chosen based on the intensity of the PCR product of the expected size, but also to minimise non-specific products. All primers were ordered dry-frozen from MWG (Ebersberg, Germany). The reaction mixture for each primer pair consisted of 25 µL of Kapa HiFi Mastermix (Kapa Biosystems, Woburn MA, USA), 2.5 µL of each primer (10 µM) and 2.5–7.5 ng of DNA template, depending on sample purity and the proportion of eukaryotic DNA in it. PCR was performed on a Mastercycler Pro S (Eppendorf, Hamburg, Germany). Cycling conditions are 95°C for 5′, 98°C for 1′, 20–25 cycles of 98°C for 20″, annealing temperature for 20″ and 72°C for 12″ followed by a final elongation step of 72°C for 1′. Annealing temperatures for each primer are presented in table 1. Gel electrophoreses (1% agarose in TAE buffer 1×) were carried out to check the size and quality of PCR products.

**Table 1:** Oligomers evaluated for their value as 18S rDNA primers.

Primer ID	Yeast 5′position	Sequencesmatched	Sequence	GC range (%)	Tm range (°C)	Reference
391f	391	26191	YGGAGARGGAGCHTGAGA	50–67	48.0–54.9	This study
550f	550	29782	GGRCMAGBCTGGTGCCAG	61–78	52.6–59.4	
563f	563	29188	GCCAGCAVCYGCGGTAAY	56–72	50.3–57.2	
574f	574	27099	CGGTAAYTCCAGCTCYAV	44–61	45.8–52.6	
574*f	574	29271	CGGTAAYTCCAGCTCYV	47–65	44.6–51.9	
616f	616	27631	TTAAAAVGYTCGTAGTYG	28–44	38.9–45.8	
616*f	616	28072	TTAAARVGYTCGTAGTYG	28–50	38.9–48.0	
897f	897	27836	AGAGGTGRAATTCTHRGA	33–44	41.2–45.8	
1132f	1132	29866	AYTTRAAGDAATTGACGG	28–44	38.9–45.8	
1132r	1150	29866	CCGTCAATTHCTTYAART	28–44	38.9–45.8	
1182f	1182	29514	AATTYGACTCAACDCRGG	39–56	43.5–50.3	
1266f	1266	29500	RGTGGTGCATGGCCGYTB	56–72	50.3–57.2	
1423f	1423	28365	AACAGGTCHGWRATGCCC	50–61	48.0–52.6	
1423r	1441	28365	GGGCATYWCDGACCTGTT	50–61	48.0–52.6	
1612r	1630	26791	ACAAAKGGCAGGGACDYA	44–61	45.8–52.6	
1626r	1644	27697	GACRGGMGGTGTGBACAA	48–67	50–54.9	
1380F	1625	27667	CCCTGCCHTTTGTACACAC	53–58	51.1–53.2	Amaral-Zettler,2009 (17)
1389F	1634	27279	TTGTACACACCGCCC	60	44.7	
1510R	1787	1699	CCTTCYGCAGGTTCACCTAC	55.60	53.8–55.9	
1391F	1770	27674	GTACACACCGCCCGTC	69	51.1	Stoeck,2010 (18)
EukB	3′	4	TGATCCTTCTGCAGGTTCACCTAC	50	57.4	
Fwd1	564	27796	CCAGCASCYGCGGTAATTCC	60–65	55.9–57.9	
Rev3	981	22175	ACTTTCGTTCTTGATYRA	28–39	38.9–43.5	
fw	366	10744	ATTAGGGTTCGATTCCGGAGAGG	50	61.4	Nolte,2010 (36)
rv	586	27376	CTGGAATTACCGCGGSTGCTG	57.1	58.3	

Oligomers evaluated for their value as 18S rDNA primers. Primer ID, where stated, refers to the ID used in the original paper. Primer position is based on *S. cerevisiae*, GenBank accession number Z75578. Primers that were too far downstream to be found in this gene are indicated as “3′”. The lowercase letters ‘f’ and ‘r’ refer to whether the primer was evaluated for use as forward or reverse. Tm was calculated using Melting Temperature (Tm) Calculation (http://www.biophp.org/minitools/melting_temperature/demo.php).

To prepare libraries for Illumina sequencing, primers 574*f and 1132r were prolonged by a handle, yielding the primer pair 5′-ACACTCTTTCCCTACACGACGCTCTTCCGATCTNNNNCGGTAATTCCAGCTCYV-3′ and 5′-AGACGTGTGCTCTTCCGATCTACTTRAAGRVATTGACGG-3′. Samples were amplified as described above, with an annealing temperature of 51°C, and cleaned as described by Lundin *et al* [45] with 15% PEG 6000, reducing the sample volume to 23 µL. To this, 25 µL of Kapa HiFi Mastermix were added and 1 µL of each of the primers 5′-AATGATACGGCGACCACCGAGATCTACAC-X_8_-ACACTCTTTCCCTACACGACG-3′ and 5′-CAAGCAGAAGACGGCATACGAGAT-X_8_-GTGACTGGAGTTCAGACGTGTGCTCTTCCGATCT-3′, where in each case X_8_ represents an 8-bp DNA barcode, so that each sample has a unique combination of forward and reverse. Reaction conditions for this second PCR are 95°C for 1′ and ten cycles of 98°C for 10″, 62°C for 30″ and 72°C for 15″, followed by a final amplification step of 1′ at 72°C. The product of this reaction was cleaned once more using the same method [45] and sequenced in SciLifeLab/NGI (Solna, Sweden) on a MiSeq (Illumina Inc, San Diego, CA, USA). For more detailed and updated protocols, see https://github.com/EnvGen/LabProtocols.

### Analysis of Sequencing Data

All sequences generated in this study and at any part of the analysis are available upon request. Raw sequences were trimmed for quality using FastX (http://hannonlab.cshl.edu/fastx_toolkit/links.html), trimming bases with a Phred score below 30 and deleting forward and reverse reads in case any of them was shorter than 150 bp. For clustering, forward reads were trimmed to 230 bp and reverse reads to 180 bp; reads shorter than this were excluded. They were concatenated and clustered using Usearch [46] at decreasing similarity levels of 100%, 99%, 98% and 97%. After clustering, the representative sequence for each cluster was separated into its forward and reverse component. Each read, or representative sequence, as described in the main body of text, were classified using SINA v1.1.13 [40]. The longest consensus taxonomy between forward and reverse reads was taken for the amplicon. Graphs and statistical analyses were produced in R, using libraries MASS, Vegan and Gplots. The taxonomic distribution of reads in environmental samples were produced in Krona (http://sourceforge.net/projects/krona). For more detailed and updated protocols, see https://github.com/EnvGen/Tutorials.

## Results

### Identification of Broad Taxonomic Range PCR Primers

**Figure 1:**
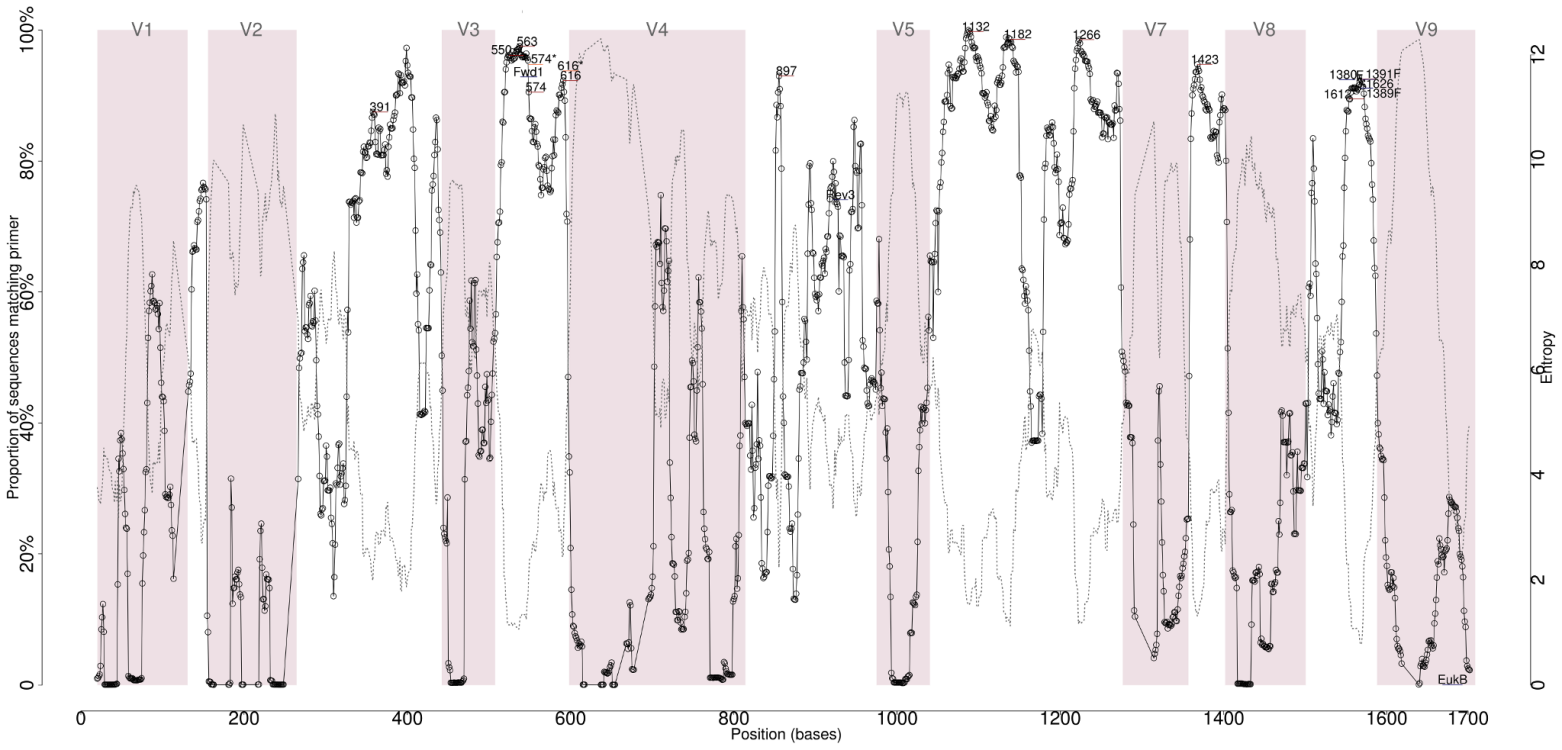
Position and coverage of candidate primers. Eighteen bp oligomers with 12 degrees of degeneracy were designed to match as many of the sequences as possible at each position of an alignment of 31,862 full-length unique eukaryotic 18S rDNA sequences using the DegePrime program. The proportion of the sequences matched by the best oligomer found for each position is depicted in black, with a line connecting adjacent points. The entropy of each position is depicted by a dotted grey line. The position numbering refers to the *Saccharomyces cerevisiae* strain FM-sc-08 18S ribosomal RNA gene, NCBI accession number Z75578. Dark red horizontal bars represent the oligomers chosen as candidate primers in this study. Primers which were later altered are marked in lighter red. Primers found in the literature are depicted in dark blue. Pink rectangles are used to highlight the hypervariable regions of the gene.

In order to identify highly conserved regions in the 18S rDNA, as well as candidate PCR primers from these, we used the program DegePrime, which, for every position of a multiple sequence alignment, outputs an oligomer of defined length and maximum degeneracy that matches an as large number of sequences as possible. Figure 1 shows the results for 31,862 non-redundant 18S rDNA sequences downloaded from the SILVA database. The potential for matching of the best primer for each position largely mirrors the entropy at the same position (R^2^ = 0.89).

**Figure 2:**
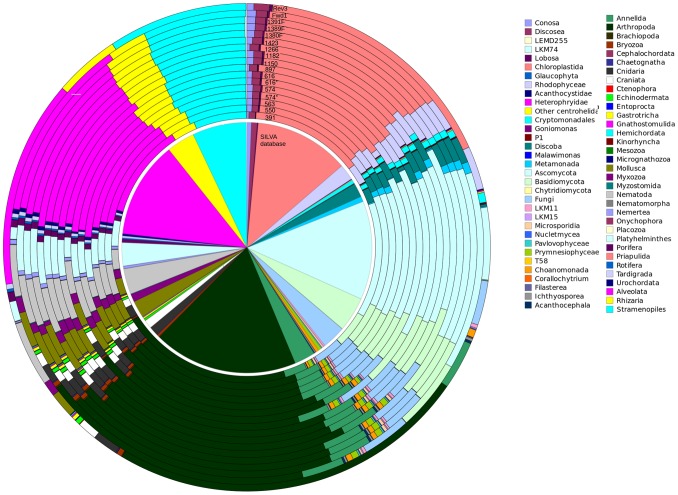
Taxonomic distribution of sequences matching candidate primers. The central circle represents the taxonomic distribution of the SILVA eukaryotic database. Each outer ring corresponds to the taxonomic distribution of sequences matching each primer candidate. Primers are marked in the figure and each colour corresponds to a kingdom or phylum as shown in the legend.

Fifteen oligomers were selected which are located in conserved regions while being flanked by variable regions. These matched 82.2–92.6% of the total pool of 31,862 full-length eukaryotic SILVA sequences (Table 1). The majority of the selected primers match all major eukaryotic lineages represented in SILVA (Fig. 2; Table S1). However, there were notable exceptions, such as the lack of coverage of some primers to the phyla Nematoda, Microsporidia or Discoba, or the Centrohelida class. To partially recover the coverage of Discoba by the candidate primer 616, another degree of degeneracy was added to the sixth base, and this alternative version was called 616*. In the case of candidate primer 574, coverage of the phyla Rodophyceae, Metamonada and Nematoda was recovered with a 17 bp alternative in the same position. Commonly used 18S annealing primers from the literature were included, and these retain the name used in the original work [17], [18]. They generally display good taxonomic coverage, although Rev3 and EukB were excluded from the analysis since they target the distal 3′ end of the 18S rDNA, which has a much sparser representation in the SILVA database (Table S1). Primers selected at this stage were combined into seven different primer pair combinations (primer pair 563–1132, 574–1132, 616–1132, 897–1423, 1132–1423, 1132–1391F and 1266–1391F), covering variable regions 4, 6, 7 and 8 and amplifying regions between 167 and 569 bp in length. These pairs were considered throughout the rest of the analysis. When used for the simulation of single-end reads, the letters ‘f’ and ‘r’, for “forward” and “reverse”, respectively, are added to the primer number to indicate the sense of the read.

### Assessing the Information Content of Amplified Fragments

In addition to matching a taxonomically broad range of organisms, a good primer pair should amplify sequences that contain as much phylogenetic information as possible. To quantify phylogenetic resolution, we performed three simulations on eukaryotic SILVA sequences which involved the *in silico* generation of amplicons and associated sequencing reads. For the simulations, we used 400 bp single reads reflecting the output of single-end sequencing strategies using 454 or IonTorrent technologies, and 2×150 bp and 2×250 bp paired reads reflecting the output of paired-end sequencing strategies using Illumina technology. Sequence reads were extracted directly downstream of each primer. Since, typically, different measures of error correction and/or OTU clustering are performed after sequencing, substantially lowering the effect of sequencing errors, we only considered error-free sequences for the subsequent analyses.

**Figure 3:**
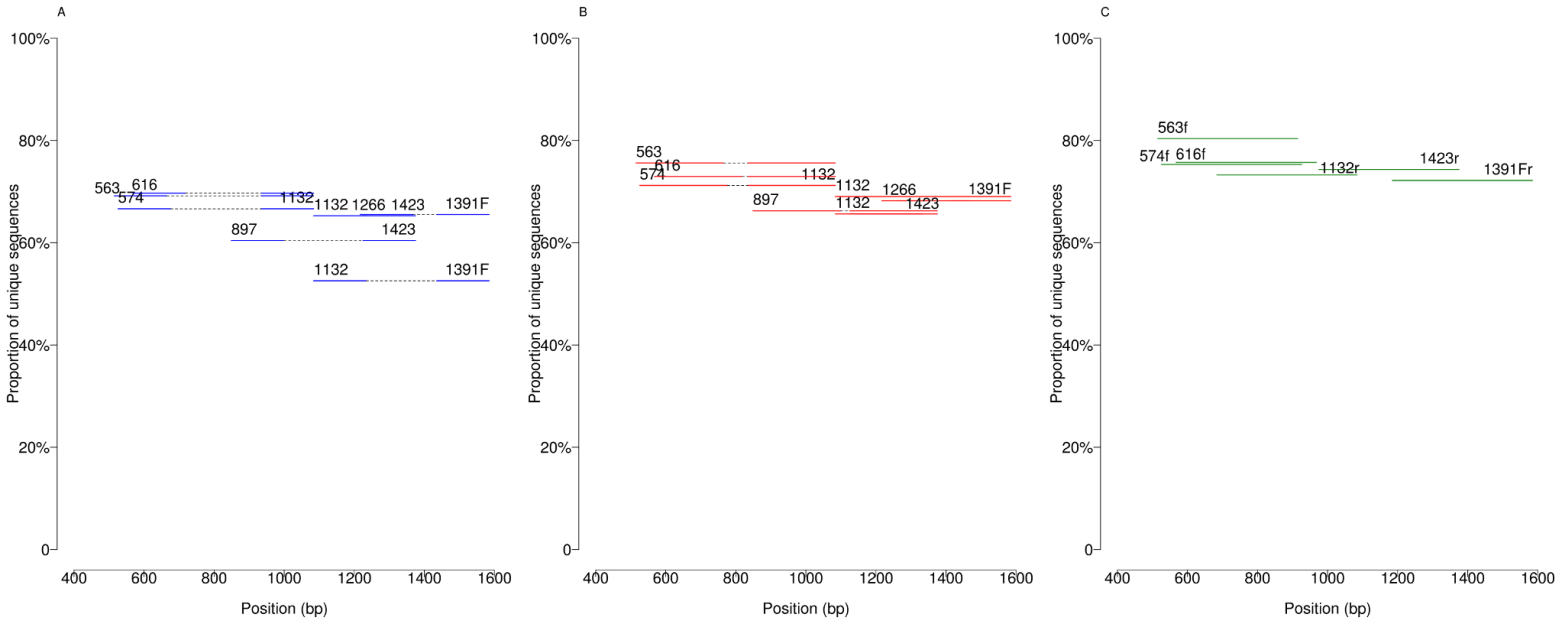
Ratio between number of unique amplicon sequences and full-length sequences. The ratio between the number of unique amplicon sequences and unique near full-length sequences (starting at primer 391 and ending at 1786), for different primer pairs and read lengths/types. (A) Paired-end 150 bp reads (B) Paired-end 250 bp reads (C) Single-end 400 bp reads. Paired-end reads are connected by a black dashed line.

The first assessment of the approach is based on the idea that, ideally, two 18S rDNA sequences that differ when comparing their full-length sequences should also differ in the sequencing reads obtained. To quantify this, we assessed how many unique artificial read sequences the non-redundant eukaryotic SILVA database would generate using the different primer pairs, read lengths and types (singlets/pairs). Figure 3 shows the percentage of unique amplicon sequences compared to the number of unique full-length sequences. Numbers differ substantially between primer pairs and read lengths, with the final number of unique reads ranging from 51.3% to 78.3% of the original number of unique sequences. Interestingly, 400 bp single-end reads have similar performance to paired reads of 150 bp or longer. Thus, this figure highlights how the choice of region can play a more significant role than the choice of sequencing technology and read length in resolving eukaryotic phylogenetic diversity by 18S rDNA amplification and high-throughput sequencing.

### Estimating Taxonomic Assignment Accuracy

**Figure 4:**
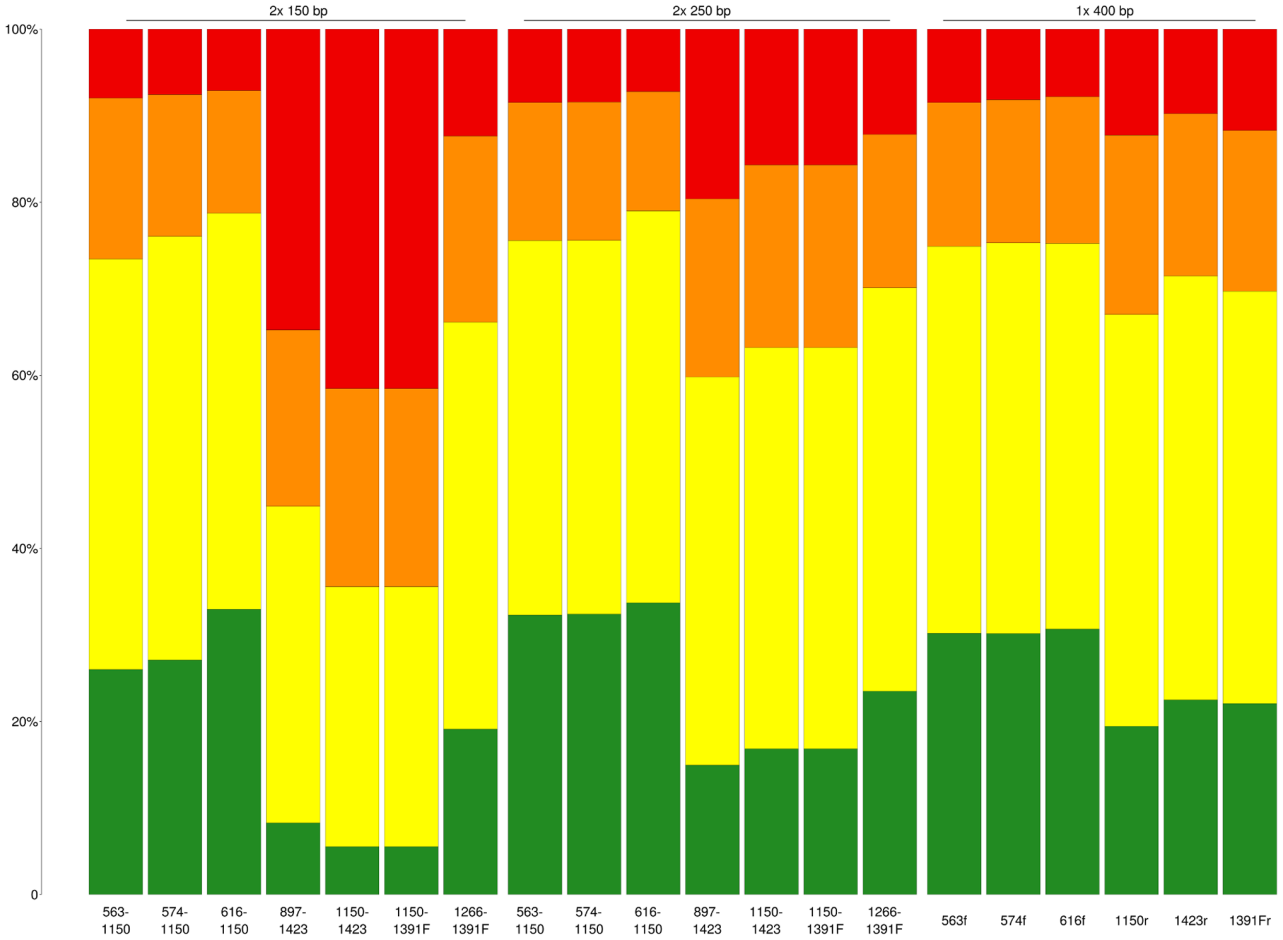
Specificity of taxonomic annotations at different taxonomic levels. Specificity of taxonomic annotations at different taxonomic levels, for the different primer pairs and read lengths/types, when requiring 99% identity to the selected match. Only instances where the selected hit sequence was annotated down to family level are shown, which were on average 78% of the cases. Matches to the correct species are depicted in green, and to the right genus in yellow. Matches to the level annotated immediately above genus are marked in orange. All other matches are considered missasignments and depicted in red.

In microbial diversity studies, obtained sequences (either individually or as representatives of operational taxonomic units) are typically compared to a database, and the taxonomic information of the best match is used to describe the sequence, provided that the match fulfils some criteria on similarity. In the second test, we simulated this situation by sub-sampling 1,000 random sequences from a subset of the non-redundant SILVA database containing only sequences matching all selected primers and with fully defined phylogeny. Error-free reads were extracted for all selected primer pairs and read lengths. The resulting sequences were BLAST searched against the entire non-redundant eukaryotic SILVA database (n = 31,862) using a threshold of 99% identity. While for the single reads we selected the highest scoring sequence that fulfilled the requirements, for the paired reads the sequence with the lowest mean of the log e-values was selected. In all cases, self-hits were disregarded. The taxonomic annotation of the query sequence was compared with that of the selected hit (Fig. 4). The accuracy of the taxonomic annotation varied significantly between primer pairs at all read lengths. While accuracy could be as low as 5.5% and 33.7% at species and genus levels, respectively, the primer pairs covering region V4 had about 30% accuracy at species level and about 75% at genus level for all read lengths. At the level immediately above genus (either family, tribe or other), at least 92% accuracy is achieved by these primer pairs at all read lengths tested. Accuracy falls somewhat at a lower percentage cutoff (Figure S1), and drops further when shorter read lengths are used (not shown).

### Estimating Phylogenetic Assignment Accuracy

**Figure 5:**
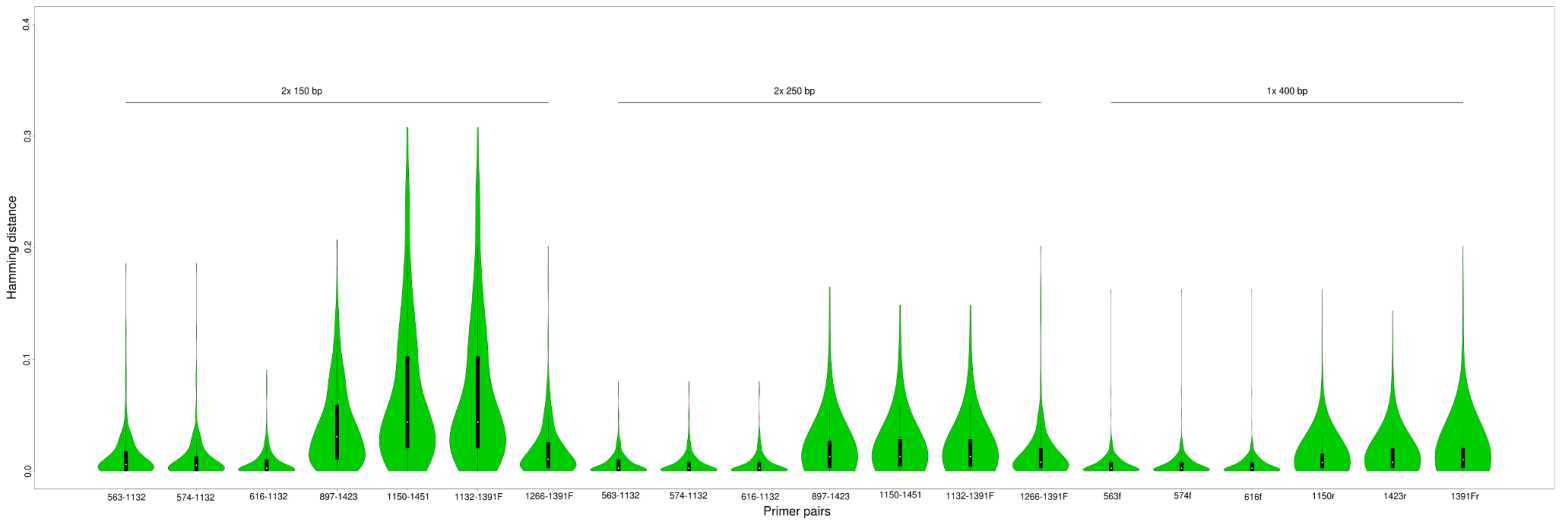
Hamming distance between blast queries and hits. A violin plot of the Hamming distance between the full-length sequence of BLAST queries and hits at a 99% identity cut-off. Inside each violin the boxplot is also depicted.

Since taxonomy is not always linked to evolutionary relationships, we also assessed the similarity of the selected best matches to the query sequences independently of taxonomy. Hence, in the third test, we used all top-scoring query-hit pairs from above and calculated the sequence distances between the full-length sequences of the queries and those of matches. Figure 5 shows the distribution of these distances for the different primer pairs and read lengths/types. With paired-ended reads of 250 bp or a single 400 bp reads, all primers have 75% of their BLAST matches within less than the 3% distance, commonly used for bacterial OTUs. The primers that allow retrieval of the V4 region have, with these read lengths, 80% of their hits at less than 1% distance and over 97% below 3% distance.

### DNA Amplification and Sequencing

As primers pairs 563-, 574-, and 616–1132 performed consistently better than any of the other pairs throughout *in silico* tests, they were selected for experimental validation. 574 and 616 were modified to increase their phylogenetic matching (see above and Table S1). Forward primers 563, 574* and 616* were thus combined with reverse primer 1132. After optimization of amplification conditions, the three pairs gave strong single bands of expected sizes for the positive control (*Saccharomyces cerevisiae*), and no amplification of the bacterial and archaeal controls. However, tested complex environmental samples sometimes yielded a second amplification product 50–100 bp smaller than the expected size. This band might represent true biological diversity, as the V4 region of the 18S gene is known to present variations in length [47]. However, the pair 574*–1132, which presented a single sharp band, was chosen for further tests.

DNA from three fungal isolates and environmental samples was amplified with primer pair 574*–1132 and sequenced with Illumina MiSeq. After quality trimming, reads were clustered at 97% similarity using Usearch and were classified using the SINA classifier [48]. Starting with 90,000 to 95,000 reads per sample, all isolates produced between 4070 and 5075 clusters, 65% of which singletons. In each fungal isolate case one cluster contained over 90% of remaining sequences and was correctly classified down to genus level. The representative sequence of each of these clusters could be matched exactly to the corresponding species in the SILVA database.

**Figure 6:**
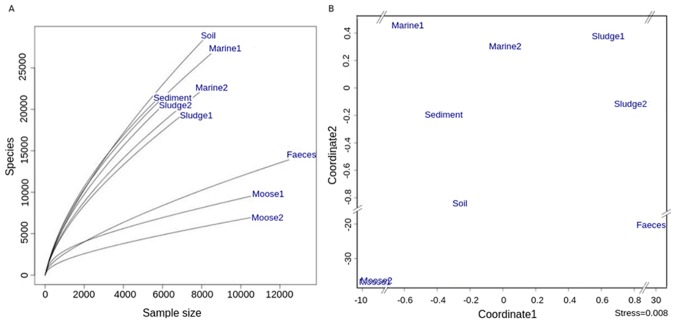
Alpha- and beta-diversity of environmental samples. (A) Rarefaction curves for OTUs at 97% similarity for environmental samples. (B) NMDA plot of the Bray-Curtis distance between 97%-similarity OTU profiles of the same samples.

The collection of environmental samples included human faeces, moose rumen content, marine water, lake sediment, wastewater activated sludge and soil. No prokaryotic DNA sequences were observed in these libraries. A first glance shows that host-associated samples are, as expected, much less diverse than samples from open environments. For bacteria, soil and sediment are believed to be among the most diverse environments, orders of magnitude more diverse than for example seawater [49]. Interestingly this was not observed for the eukaryotic communities analysed here (Fig. 6A, Table 2). As might be expected, samples from similar environments have a somewhat similar community composition, with the predominant difference between communities being the separation between free-living and host-associated (Fig. 6B).

**Table 2:** Estimators of alpha-diversity for the environmental samples sequenced.

Sample	Shannon	Pielou	Chao1
**Marine1**	4.128	0.6150	1109.4
**Marine2**	3.605	0.5690	716.0
**Soil**	3.959	0.5760	1272.3
**Sediment**	3.674	0.5740	819.2
**Sludge1**	2.645	0.4370	628.5
**Sludge2**	2.984	0.4760	692.7
**Moose1**	1.963	0.3270	270.1
**Moose2**	0.712	0.1440	189.1
**Faeces**	1.315	0.2950	144.3

**Figure 7:**
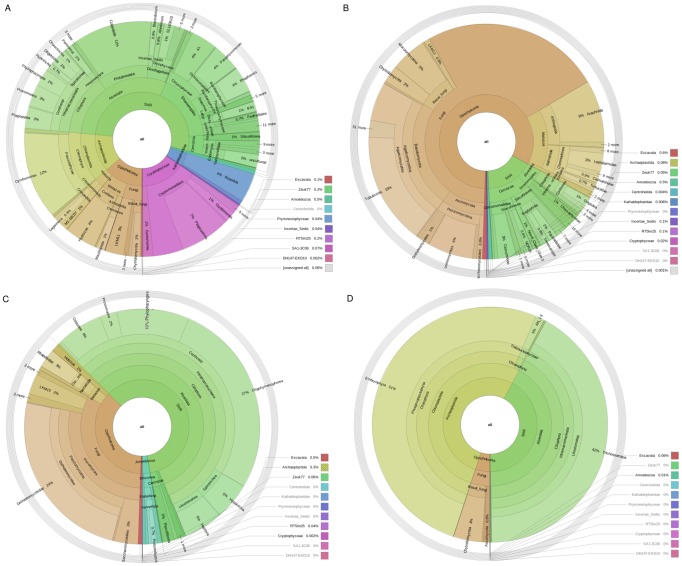
Taxonomic classification of selected environmental samples Taxonomic classification of selected environmental samples. (A) Marine water 1 (B) Soil (C) Wastewater sludge 2 (D) Moose rumen 1. An interactive HTML version of these plots and of the other environmental samples at deeper taxonomic resolution can be found as File S1.

Marine 1 emerges as one of the most diverse samples (Fig. 6A, Table 2). This sample was collected close to shore in a closed bay, unlike Marine 2, which was collected in open sea (both central Baltic Sea). While Marine 2 is dominated by the SAR group (Stramenopiles, Alveolata and Rhizaria), and had 53% of its sequences matching to Dinophyceae, Marine 1 presents a more diverse community, including significant amounts of Chloroplastida and Opisthokonta (Fig. 7A, File S1). Also remarkable is the 7% of sequences in Marine 1 assigned to relatively unknown phylum Katablepharidae [50]. Most previous studies of eukaryotic life in soil have focused on fungi. Our observations are in good agreement with these, such as a large dominance of Basidyomicota and Ascomycota, with much smaller fractions of other phyla such as Chytridiomycota [51]. However, we observe a larger proportion of the deep branching fungi LKM11 and LKM15 in our soil sample (Fig. 7B). The larger proportion of unclassified reads in the lake sediment as compared to the soil sample suggests unexplored microbial diversity, although the total alpha-diversity seems similar (Fig. 6A, File S1). By far the largest group in both wastewater samples was the subclass Peritrichia (Fig. 7C, File S1), which is known to play an important role in wastewater community dynamics [52]. The second largest group observed, the Cercozoa, has been observed in wastewater [53], [54] and is also found in marine and fresh water environment samples. Interestingly, Metazoan predators such as Rhabditida and Rotifers were also found in these samples, indicating a complex, multi-layered ecosystem.

In the host-associated communities, little or no host DNA was amplified. For the moose rumen samples, a significant proportion (4 and 53% respectively) of the 18S sequences were derived from feed (Chloroplastida). One of the rumens also contained substantial amounts of fungal sequences (5% of the sequences). The remainder was dominated by SAR, of which the rumen ciliate Entodinium [55] made up a large proportion (Fig. 7D, File S1). In the human gut, interestingly 85% of sequences were classified as Parabasalids (File S1), for which both parasitic and commensal members are known.

## Discussion

We defined a set of primer pairs suitable for massively parallel sequencing of the eukaryotic 18S rDNA which covers different variable regions of the gene. Primer pairs 563–1132, 574–1132 and 616–1132 cover regions V4 and V5; 897–1423, V5 and V7; 1132–1423 and 1266–1423, solely V7 and 1132–1391F and 1266–1391F, V7 and V8. Our analyses suggest that regions V4 and V5 are the most information-rich. By using the paired-read information obtained with, for example, Illumina or IonTorrent sequencing, one has the possibility to combine these two regions (combining forward primers 563, 574* or 616* with reverse primer 1132), and the sequences generated are as discriminating as the longer reads achievable with 454 pyrosequencing (Fig. 4 and 5). As both Illumina and IonTorrent sequencing have a much lower cost per read, this allows for deeper sequencing efforts without loss in data quality.

While primer pair 616–1132 performed consistently well in all tests, forward primers 616 and even 616* have the disadvantage of missing some important groups of organisms (Table S1). While the added degeneracy of 616*, as compared to 616, partially rescued its capacity of binding to Discoba, both forms still fail to amplify most fungi, Microsporidia and Metamonada (including *Giardia* sp.). In contrast, the modified primer 574* fully recovers binding to Metamonada, but still fails to fully match to Acantocephala and Microsporidia. Primer 563 is one of the few of the designed primers that doesn’t miss any major group of organisms in our detailed analysis (Table S1). It can be suitably combined with primer 1132, which also binds to all major eukaryotic phyla.

It is important to notice that, while primer-binding is a strong limiting factor to the amplification of DNA tags from environmental samples, it is by no means the only one. The presence, structure and composition of a cell wall plays an important role in the efficiency of cell lysis and DNA extraction. Humic and fulvic acids, as well as pigments and cations, inhibit PCR in a template-specific manner, where the length of the amplicon and the melting temperature of the primer play important parts [56]. The V4 region we propose here can present variations in size of over 100 bp [47]. Furthermore, when dealing with degenerate primers, the annealing temperature is not the same for every variant of it, and therefore to every template present. The length of the amplicon can also affect the efficiency of cluster detection in solid-phase sequencing-by-synthesis, with shorter DNA amplicons producing sharper clusters and better results.

Due to the richness of the data obtained by combining variable regions with paired-end reads, the primer combinations and BLAST parameters used produced median distances between the full-length sequences of queries and hits well below the 3% commonly adopted for binning bacteria into operational taxonomic units at the species level [57] (Fig. 5). However, the taxonomic assignment of reads is still generally only trustworthy at the genus level or higher in the taxonomy (Fig. 4), with 79% accuracy at the genus level for the best primer pair using 2×250 bp reads. It is further important to note that the sample taken as query for the BLAST analyses contained only species of known phylogeny, to allow for their comparison. On a sample from a poorly characterized environment (as our environmental sequencing showed), most sequences will probably lack a database relative within close enough sequence distance to infer taxonomy at the genus level.

The 79% accuracy in genus-level taxonomic assignment observed is considerably below what was seen in a similar analysis conducted on the bacterial 16S rDNA, where 94% of genus assignments were correct when reading only 59 bp from variable region V6 [58]. This discrepancy is probably due both to intrinsic characteristics of eukaryotic organisms and to how their taxonomy has been defined [17]. Since eukaryotes are more morphologically distinct than bacteria, they tend to be more finely classified. Organisms with genome differences small enough to be considered to belong to the same species in the bacterial world can be assigned to different genera in the eukaryotic world [59]. This most likely contributed to the relatively poor performance of classification of the yeast isolates. All isolates belong to the Saccharomycetales order, which includes 1050 sequences from 31 different families in the SILVA 111 database. This abundance of near matches can affect the performance of the classifier, which outputs a conservative class-level classification. For fine-scale phylogenetic analysis of fungi it may be necessary to use more fast evolving sequence regions such as the internal transcribed spacer [60].

The very large variation in gene copy number limits the use of 18S rDNA to a semiquantitative approach [61]. The relative proportion of an OTU can be compared between different samples, but no conclusion can be made on its absolute abundance based only on sequencing read number. One way of side-stepping this issue is to extract RNA from samples and prepare cDNA libraries. This approach reduces the phylogenetic copy number variation, privileging instead the amplification of cDNA fragments from highly physiologically active cells, with many ribosomes [62].

Despite these concerns, the sequencing results presented here show that a meaningful qualitative picture of the eukaryotic microorganisms of complex environments can be obtained by deep sequencing of the V4 region of the 18S rDNA gene. The relationships between OTU defined by the V4 region and morphospecies is currently under investigation by the CBOL Protist Working Group [63]. This working group will also investigate the suitability and necessity of sequencing lineage-specific gene tags for protistan identification.

Microbial eukaryotes are a fundamental part of all ecosystems, but are often overlooked in microbial research, due to both the relative difficulty in studying them (when compared to bacteria) and historical biases [64]. Here, we present PCR primer pairs that can help bridge this gap, by allowing minimally biased and information-dense 18S rDNA amplicon sequencing.

## Supporting Information

Figure S1 Specificity of taxonomic annotations with 95% identity cut-off. Specificity of taxonomic annotations at different taxonomic levels, for the different primer pairs and read lengths/types, when requiring 95% identity to the selected match. Only instances where the selected hit sequence was annotated down to family level are shown. Matches to the correct species are depicted in green, and to the right genus in yellow. Matches to the level annotated immediately above genus are marked in orange. All other matches are considered missasignments and depicted in red.
(TIF)

Table S1 Proportion of sequences matched by each primer in each taxon. Amount of sequences from each kingdom or phylum in the original SILVA database that matches each of the candidate oligonucleotides. The percentage of the population matching is also indicated.
(XLS)

File S1 Taxonomic classification of all environmental samples.
(HTML)
