# Correction: Systematic Design of 18S rRNA Gene Primers for Determining Eukaryotic Diversity in Microbial Consortia

**DOI:** 10.1371/journal.pone.0117636

**Published:** 2015-01-30

**Authors:** The PLOS ONE Staff

There is an error in the Materials and Methods section in the first sentence of the third paragraph under the subheading “DNA Extraction, Amplification and Sequencing.” The reverse primer sequence for the first PCR reaction is incorrect. The correct reverse primer sequence is: 5'-TGGGCTCGGAGATGTGTATAAGAGACAGCCGTCAATTHCTTYAART-3'
