# Supplementary figures and images for: Systematic Design of 18S rRNA Gene Primers for Determining Eukaryotic Diversity in Microbial Consortia
Source: PLoS One. 2014 Apr 22;9(4):e95567. doi: 10.1371/journal.pone.0095567 (PMC3995771; doi:10.1371/journal.pone.0095567)

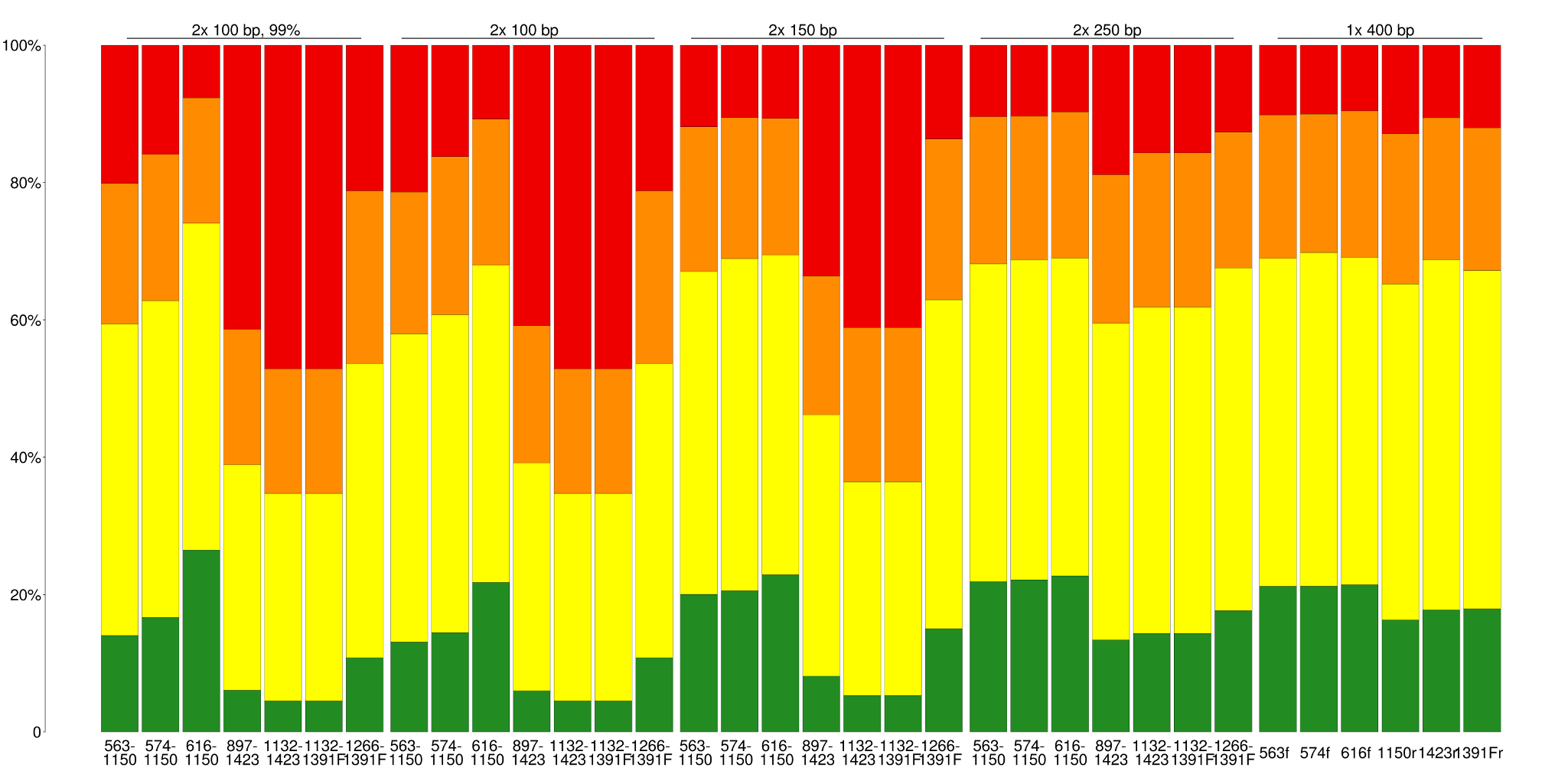

Supplement: Figure S1 — Specificity of taxonomic annotations with 95% identity cut-off. Specificity of taxonomic annotations at different taxonomic levels, for the different primer pairs and read lengths/types, when requiring 95% identity to the selected match. Only instances where the selected hit sequence was annotated down to family level are shown. Matches to the correct species are depicted in green, and to the right genus in yellow. Matches to the level annotated immediately above genus are marked in orange. All other matches are considered missasignments and depicted in red. (TIF) [file pone.0095567.s001.tif]
