# Supplementary material for: Systematic Design of 18S rRNA Gene Primers for Determining Eukaryotic Diversity in Microbial Consortia
Source: PLoS One. 2014 Apr 22;9(4):e95567. doi: 10.1371/journal.pone.0095567 (PMC3995771; doi:10.1371/journal.pone.0095567)
Supplement: File S1 — Taxonomic classification of all environmental samples. (HTML) [file pone.0095567.s003.html]

Javascript must be enabled to view this page.

members
magnitude

Human1
Marine1
Marine2
Moose1
Moose2
Sediment
Soil
Waste1
Waste2

117823851098057410860210833154491769856445658925

3433

3433

3

3

2

2

3427

6

2111

54

1

12099574244138

303

29

1

3

39013455

34133112

458333

291

1000079948036474424368320

99480347392368318

3

3

99480347392368315

88480345392368315

449126334258

6

6

6

1

3

2

443126334258

443126334258

2

2

824799362663457

6256

6256

76479936103457

1216

1216

184227

8

2

84222

534799345443

534799345443

427

427

112

112

112

5

1000076427272

25

25

100007642272

79035282

48093

11642

36451

21596

7

981

1

1021

87

444

1

129836410

24672

3471

5108624

53211

456141311106140310181569

181

7910536511

5

5

2433

17

1010

1010

71

2

1

1

3

1

79725

3724

761

364133109961529651258

22096337

294254

294254

20283

17

1

2

3641338976152869921

3421338976151721732

28670

2238395

24222021247115

431612361146

1148189

99

1102158

32

22

22

13111117853299

13111117853299

131194152298

422

15452291

13157

1120

193

193

27

27

11611

112

411

1

1

18610974175857320472457325924162

18610974175857320472457275919153

1781504455735470244361533

3510

3510

1781154455735470244361523

178984455735470244361523

178984455735470244361523

10

1788844557354702251523

1

1

1

17837557354702251523

13753962181

13753962181

1

1

1

355314215

12

1

1

1

153

177503394484251522

535

534

12

12

56

56

1

1

24724

2

1

844

1

52

3

3

4

3

3

21

2

1

69

65

4

91

91

1

2361

2361

69522301511

1

230

28287078

2

1

410132683

1

3282

1

15

186

127

3028064816

270929716

1

3

3513

596

2

1171532

1

9

9

15038

15038

2521183

2

2521163

6853772

2

51772

1

2956

30

8

2

11

11

103100755581016

1

110070543101

1024156

1196

1

42

12

1515

12

2

2

1

877

867

22

11

2

616

4186

2

2

27

27

2953

1

1

131

1

1

1

1

78

3

4

4

13

13

81082417141377225284374120

72140712222

61

127

2

32

213761

110315977

2

96773

110217204

73420713772139642475

1

1924

27

2

613301738

1

12

1

1

4

1

445

282

1

416

2

11

1

19

526214

2

2

54

3

49111952823

491

10

1

12

3

11

401

65

9

30

41442

1

512722

78

1

1

2

3543

1

3

6

10

1

1

106

5

205

3

1

20816

559

59

5

24

944724708004578210334519299125125823235198

62539650509456521032621004122344656633458

1967795881601455

15

15

1318955153131

2545322110

4521

384

3

163039920

982

1262

16

1091

11

913518711

913518711

405728706337517

78188430721

1

1

78188230721

27471

715

715

303

21

141336

24852682625712

125461

4353183

2

1923181

24282

186

12

128545334

15817

3

12

3517

110544117

171

3369

8753998

5

14

4

2

2

16

1

471186412

371186412

100

4751922724

12734

3684

2

8

7

22

1

3

2

12

12

375

214312

214312

39813539

4155524

102

181316

36

1716

18111

148

22982766442787

22982766442787

9827612156

982758118

72207

11

43

1

26715

3

3

31045

31

314

37

32124

23

23

3211

321

22222527

22222527

5114071982345546103235639620254653933366

100881659980

100881659980

5

13501

1

9258168880

1

63

5103991981545546103234639019664653033286

3323031114

3323031114

27582437726104162214744390227065

12464877118546211256

12464877118546211256

2318212116512

6726966

56582111

19979991621

653220212

25891502613817115421396

21941072491164219275

874578

20188916191384328185884

19021116191311128005870

222

61548

7481116213171241161

461522095379

116787321814

114

176

5542121

161

5743322

337228121

1144188117111294222393675

114378117110265422383672

383

114274982010756

7

6

114139743029

13152992

147

553525561114540

1161411

15211

1245

284

257

27

177213

11

177112

3384

1

33

2

81

3673739511492513709015853

673562437021438

44272114

1012

535581411207

12

11

11

21

1

713

11

24

1

11

2

25154511031703687115800

1021

33174518

85

1

181

1

222215810

264

144

51

65

137148823359215250

112

1

2

47123311410

111

241183

4

2148

1242121

116

13

78

114213164552010312064536211473176

356455201031165217121

2

1

121

9

1

6

45087884642110

1

334114

17

43114635

11071310459334511353175

311472613251

2711231084416

11

5514513

3911

744961300

616628

55

51823032

41163561

1464831157

226421412010382012914803031

25683811034791242632990

21

6

8637810613642152901

1

23192829173026

7

11

31

21335

6643

1

7

1

110

5923711121411

1211

38

21237111202

11448003384114

135

338

50381

10906033383114

871127022216

1

11

543611

27312452115

543

2392

239

788381

4284220761217148526108831270

4284220761217148526108831270

189

133194885

3332

12130

92754233

43

309632823

98976116831161465250

212875

356846151122

121285

121285

2222612101417112

26224413

1164

116

4

256449

2142

63

1

21132

11

12261

1245

1211019572

30

30

111955

111955

3

1

6412

811

10

10

10318

10318

18113191222620

38322741

311017952219

498457783235427

1

1

1023199561042013166917915

121

121

718941262659

144001155684179556

3829771631667

225271219160125

554242331335

5125341143

5125341143

21818969712811138396

1841166919132997287

1841166919132997287

347352149141109

2273511471405

12121104

38263061071

30

1

13511392358413

193

1351113193417

241285

120331

38920316131104

1576110931

1911

26

40

1251

1562183

1560

2183

14

1023

16

16

5113602911

5113602911

216

142

315044180841247175441752783470

1858791322

2

1

1858491322

423400114

12341

350

32

1

4362156608511319

1205411

11

47210

391416217

25

58

1

1

832

2731

6

33

85236093

11622

3169

983441

583441

40

184142372919

16

2810

1510

13

1610712

271

36122419

36122419

5910134

1821

2

4110111

635584113

12594588236

3136

11111

753

12383167196

138

13151

21201

19

119233

5

11111

17087792663662248761328248

256203148910

23

30851591961118

365349253215

45349253215

5103

1329158763635234221366

11671163631309546

3117982

223

2814

1

3

1121361219

3347610333151189209

10814213705318

9913213535318

318357512080136191

56211

5

5

11815911

1357333681127539214281

1766987115

12

1

2

2

2

1752987115

1032

1785

7

68122113

5

63

34963361127137414266

3082467161136

2

1184

28142

12

1111

29811

1

6

2

140122

31151

1311

4142894111036114260

197

1

1

65362311

22014

4246

15515

43

3311

1

221515

135170215146

11

1

217760411773128

1

111717

5414921611

1

6918512042

51361

51361

1111

11020

11020

11020

4

3

3

1

1

93045526195405

1825

33

1522

408279405

61124419186

47216818184

3820

402612

2110

13

244451

244451

29337

29337

5

40

7

116

2

111

31

2

4

47311

4464201

235188114335315615

235188114335315615

106181973426

26

356161

4510282425

1993021210

117

16

2

11

11

1991561210

32

306711884

112184

84

1121

117

117

165

18

18

15013276278926

1295463826191

1176963210

12651

68

458251

1902951

11

8631

1222652

28

153

587813

118562691

58

1762011469177854232491995063356480521611

26250491342366119944101847

11

11

11

64518

64518

64518

26

2312

9921

921

9

9

9

10793972270

10793972270

10793972268

2

12578

12578

12578

3753921

3753921

2

375351

4

36547623641563

36538903641563

452

32

14

22

1

27

22

4

197123

3

7

15

2

17

2556

341

2323

360

689110

15283031543

872

5

541

289

17

4

15

9104931171191144

321517

171

15517

1006311

1

1005311

91166011144

91166011144

2531311443

2531311443

2531311443

18

18

16

2

4863401

21021030121965492893584

867391112435223654

867391112435223654

11176357073111973321

1103216679311970122

7527064452

1

341110012186742

626734864

196853459223

23621

14419282803199

11419282803199

3

554236185633132

631015177

631015177

127

1

2

17358427061454212121768550773150219179

11611993210

11611993210

11611993210

17349101769091741168727239316406

128757569168108566717114460

112114

112114

112114

13

3

1

1

830

406447903371150114274

3

3

3

3

101

101

101

16

85

92467

92467

4

4

92463

40231466319490114228

28245225111114175

28245225111114175

12269669437953

1195773

313

13

331275

1214630563

3

1191413250

79170251

1

1

1

1

1

113

113

113

113

113

11325163210434213

56311616

56311616

12

5621596

1

1

1132120927327

1132120927227

1132119726927

1154692553448116678

11373

154

154

154

154

1733714193406836013221946

1733714193406836013221946

1733714193406836013221946

1

1

13

13

165334412712193

34

16533449312193

32

3

2

11

11

32252762272

32252622241

7

29

7

2

2

2

173233106131123911

352

1732131061311224

2212322861646

222322641458

19109

56

1323

17014

11614

54

76

12

2

61

1

11398345428923116572459279

11398345428923116572459279

11398345428923116572459279

1106134442672309718684960

4

4

3

3

22722337511036

1101146

1158933

13

13

23859156897201648

174288118315

174288118315

8167222341099318140456389

5120818310

5120818310

21

51311825

125525018

5212

5

212

24

24

2436

2131

22

141

72

1

1012

1

1

25

3

19

1

15

36

1

316677211097517887456361

21166510100157440314

1

29

1431

148

17293122

71

1011

22

15121110875177291647

463

323

111832

7

1

2

17

4

1

1

47

12

34

2

5

1

19

18

1

177

1

5

13

1

2

85

151

1

2

4

1

1

1

12

12

1

5

2309

60

2

48

15251

9

3

23

76

112315

9

6

9

222

14

1

7753

31

2

1

1

7

221612611133764252277

1537

5

11

5

4

26

1
